# Acceptability of Mobile Health Interventions to Increase Diabetic Risk Factor Awareness Among the Commuter Population in Johannesburg: Descriptive Cross-Sectional Study

**DOI:** 10.2196/12600

**Published:** 2019-09-20

**Authors:** Alex Fischer, Martha Chadyiwa, Ndumiso Tshuma, Vusumuzi Nkosi

**Affiliations:** 1 Department of Environmental Health Faculty of Health Sciences University of Johannesburg Johannesburg South Africa; 2 Best Health Solutions Johannesburg South Africa; 3 Environment and Health Research Unit South African Medical Research Council Johannesburg South Africa; 4 School of Health Systems and Public Health Faculty of Health Sciences University of Pretoria Pretoria South Africa

**Keywords:** mHealth, diabetes mellitus, noncommunicable disease, South Africa

## Abstract

**Background:**

Developing countries are experiencing a shift from infectious diseases such as HIV and tuberculosis to noncommunicable diseases (NCDs) such as diabetes. Diabetes accounts for more disability-adjusted life years than any other NCD in South Africa, and research has identified a number of preventable risk factors; however, there is not enough evidence from lower resource settings as to how best to disseminate this information to the population. Today, 90% of the world’s population lives in mobile phone coverage areas, and this provides a unique opportunity to reach large populations with health information.

**Objective:**

This study aimed to investigate how potential mobile health (mHealth) platforms should be paired with diabetes risk factor education so that at-risk communities are empowered with information to prevent and manage diabetes.

**Methods:**

A Likert-style survey was distributed to commuters in the City of Johannesburg in July 2018 that explored participants’ background characteristics as well as their knowledge and awareness surrounding diabetic risk factors (such as exercise, smoking, and hypertension) and their comfort level with various information delivery methods (such as WhatsApp, short message service, and email). The grouped variables from diabetic risk factors and information delivery methods were described with mean Likert scores and then investigated for relationships with Spearman Rho correlation coefficients.

**Results:**

Background characteristics revealed that the self-reported prevalence of diabetes was twice as high in this studied commuter population than the national average. WhatsApp was the most favorable mHealth information delivery method and had a moderate correlation coefficient with diet and nutrition (0.338; *P*<.001) as well as a weaker correlation with physical activity (0.243; *P*<.001). Although not as robust as the WhatsApp correlations, each of the other information delivery methods also showed weaker, yet statistically significant, relationships with one or more of the risk factors.

**Conclusions:**

The elevated self-reported diabetes prevalence reinforces the need for diabetes risk factor education in the studied commuter population of Johannesburg. The most feasible mHealth intervention for diabetic risk factor education should focus on WhatsApp messaging while also offering content across other mHealth and traditional platforms to remove barriers to access and enhance the user experience. The content should emphasize diet and nutrition as well as physical activity while also incorporating information on secondary risk factors.

## Introduction

### Background

In developing countries, advances in maternal health and infectious disease treatments are causing a shift in the burden of disease from communicable diseases to noncommunicable diseases (NCDs). In South Africa, 5.5% of the population (2.28 million people) is affected by diabetes, and another 9.9% of the population is described as prediabetic [1,2]. The prevalence of diabetes in low-income communities is estimated to be 7.1%, as these communities are more susceptible to diabetes because of specific risk factors [3]. These risk factors include unbalanced diets because of food insecurity, cultural influence on food and body-image perceptions, lack of physical activity, as well as a general lack of knowledge surrounding diabetes [4,5]. These risk factors are often preventable and have been well defined in literature; however, there is very little information regarding how these communities comprehend the risk factors and how they would like more information on them.

With the recent advances in technology over the last two decades, developing countries are also experiencing a surge in mobile penetration; the cellular market in Africa is estimated at over 1 billion subscribers, and there are more mobile phone users in sub-Saharan Africa than the entire United States [6]. In South Africa specifically, mobile penetration has reached 68%, and up to 90% of smartphone users regularly use at least one app-based messaging service, such as WhatsApp or Facebook Messenger [7]. This provides a unique opportunity to distribute health information to large populations, and in 2015, the South African National Department of Health introduced the South African mHealth Strategy 2015-2019. This strategy was initiated as a way to promote and regulate the use of mobile health (mHealth) initiatives to strengthen health care [8]. Since then, many mHealth interventions have been successfully initiated; however, majority of the interventions support maternal health or HIV programs and very few of the interventions focus on NCDs [9-12].

There are, however, several diabetes-related apps available from Web-based stores such as Google Play, but the majority of apps focus on tracking blood sugar levels, with very little attention being directed toward prevention and education, as stressed by clinical guidelines [13,14]. Messaging and internet interventions have also been implemented, and evidence suggests that they might change behavior by promoting fruit and vegetable intake as well as increased exercise [15,16]. These apps and other mHealth platforms provide proof of concept, but many of these studies are pilot projects with small sample sizes or high elements of bias [17,18].

### Objectives

Despite successful mHealth interventions showing proof of concept in the local setting and diabetic mHealth interventions showing effectiveness elsewhere, there is not yet a strong enough body of evidence to guide the development of mHealth interventions for South Africans at risk of diabetes [19]. To ensure that new mHealth interventions have high acceptability, these knowledge gaps must be filled, and this can be done by incorporating end users into the design process [20,21]. By surveying end users, this study aimed to investigate how potential mHealth platforms should be paired with diabetes risk factor education so that at-risk communities are empowered with information to prevent and manage the disease.

## Methods

### Setting

A descriptive cross-sectional study was conducted with a convenience sample of commuters from the City of Johannesburg. The public space surrounding the Noord Street Taxi Rank was selected as the venue for this venue-based intercept study because it is one of the busiest mini-bus taxi hubs in Johannesburg. This hub serves as a transfer station for commuters coming from nearby townships as well as the starting point for many residents of the Central Business District. The mini-bus taxi system is used primarily by individuals with lower socioeconomic status, as it is the most affordable means of transportation in the city. The commuter population was, therefore, studied because it offers an opportunity to engage with a high volume of low- and middle-income individuals on a neutral territory [22,23].

### Survey Design

Standardized surveys for diabetes risk factors are all associated with quantifying a persons’ risks, not their perceptions or openness to learning about risks, so a new data collection tool was needed for this study. To explore participants’ perceptions surrounding diabetic risk factor education as well as their comfort and openness toward using different information delivery methods, a new unvalidated survey was created by the authors. This new survey used groups of perception-based questions, which are utilized in human-centered design by many consulting firms such as IDEO [20,21]. This form of data collection is common practice in many industries, and in sub-Saharan Africa, digital financial services incorporate this into the development of mobile banking apps [24].

Categorical demographics questions were used to describe the sample population with self-reporting, whereas a Likert scale from 1 to 5 was used to explore perceptions surrounding diabetic risk factor education and information delivery methods by creating grouped variables from 3 to 4 related questions. The Likert scores for diabetic risk factor represented the participants’ interest in learning about that topic, whereas the scores for information delivery methods represented the participants’ willingness to use each intervention.

The complete list of variables is as follows: *demographics* (gender, age, ethnicity, marital status, education, employment, economic status, residence, household size, medication, diabetes [informed by doctor], high blood sugar [informed by doctor], risk of diabetes [informed by doctor], weight, and body type), *diabetic risk factors* (diet and nutrition, physical activity, smoking, alcohol, hypertension, and medication use), and *information delivery methods* (television, radio, newspaper, short message service [SMS], WhatsApp, internet access, email, mobile apps, social media, and face-to-face). The complete survey is available as Multimedia Appendix 1.

### Data Collection

Before data collection, the survey was reviewed by 2 public health specialists and a statistician and then pilot tested for usability. A sample size of 400 was required to obtain results with a maximum discrepancy of 5% and a CI of 95%. This was calculated by a statistician with a CI-based formula. Data collection was done by trained field workers who used convenience sampling to distribute the Likert-style survey to consenting individuals surrounding the Noord Street Taxi Rank between July 2, 2018, and July 6, 2018. Participants were included in the study if there were older than 18 years and provided informed consent, whereas participants were excluded if they were younger than 18 years or did not want to participate for any reason. The data were then entered into Microsoft Excel V16.16.2 (Microsoft Corporation), and an independent third party reviewed the entries for accuracy. SPSS Statistics V 21 (IBM Corporation) was used for data analysis, and after the data were imported, variables were created and divided into 3 categories: demographics, diabetic risk factors, and information delivery methods.

### Data Analysis

Descriptive statistics were used to analyze the demographic characteristics and explore them as categorical variables. Subcategories were then described with frequencies and percentages.

For diabetic risk factors and information delivery methods, grouped variables were initially tested for internal reliability with Cronbach alpha statistics, and groups were considered reliable with an alpha coefficient greater than .7 or a mean interitem correlation above 0.3 [25,26]. The medication use grouped variable from diabetic risk factors was not considered reliable, and in information delivery methods, the question “I have access to a cell phone” was also removed from the SMS original grouped variable, and a new variable, SMS, was created, which was reliable.

The internally reliable grouped variables were then explored with descriptive statistics to identify means and SDs. Spearman’ Rho correlation coefficients were employed to identify relationships between each diabetic risk factor and each information delivery method. A coefficient approaching 1 represents strong positive correlation between 2 variables, whereas a value of 0 represents no correlation at all [27].

### Ethical Consideration and Approval

The University of Johannesburg Research Ethics Committee granted approval on April 17, 2018, with the National Health Research Ethics Committee registration no: REC-241112-035. No incentives or compensation was provided to any of the study participants.

## Results

### Descriptive Statistics

A total of 364 predominantly black individuals completed the survey, and 230 (63.2%, 230/364) of these participants were male. The mean age was 35 years, and the participants’ ages ranged from 18 to 65 years. The majority of respondents had completed at least grade 12, and one-quarter of all participants stated that they were unemployed. When asked to describe their economic status, 123 (33.8%, 123/364) self-reported as below average or poor, 190 (52.20%, 190/364) as average, and 51 (14.0%, 51/364) as above average or wealthy. Half of the participants were married or living with their partner, whereas 90.1% (328/364) stated that they share a household with at least one other person.

One-quarter of all participants stated that they took some sort of medications on a regular basis, and 51 (14.3%, 51/364) participants stated that they had been told that they were at risk of diabetes from their doctor. With respect to diabetes diagnoses, 45 (12.4%, 45/364) participants self-reported that were told by a doctor that they had diabetes, and 46 (12.6%, 46/364) participants stated that they were told by a doctor they had high blood sugar. When asked to self-report their described weight, 42 (11.5%, 42/364) participants were below average, 283 (77.8%, 283/364) were average, and 38 (10.4%, 38/364) were above average. Participants were also asked to self-report their described body types, and 161 (44.2%, 161/364) participants self-reported as skinny or small, 167 (45.9%, 167/364) as average, and 36 (9.9%, 36/364) as overweight or obese. A total of 18 (5.0%, 18/364) participants did not provide an age, and 4 (1.1%, 4/364) participants did not respond to weather they had been told by a doctor that they were at risk of diabetes. All other background characteristics had less than 1% missing data. Table 1 summarizes the demographic characteristics of the study participants.

**Table 1:** Background characteristics of participants.

Characteristic		n (%)^a^
**Gender**		
	Male	230 (63.2)
	Female	134 (36.8)
**Age (years)**		
	18-24	99 (27.2)
	25-30	94 (25.8)
	31-40	80 (21.0)
	>41	73 (20.1)
	Missing	18 (5.0)
**Ethnicity**		
	Black	325 (89.3)
	White	5 (1.4)
	Colored^b^	23 (6.3)
	Indian or Asian	4 (1.1)
	Other	4 (1.1)
	Missing	3 (0.8)
**Marital status**		
	Single	156 (42.9)
	Living with partner	75 (20.6)
	Married	110 (30.2)
	Divorced or separated	18 (5.0)
	Widowed	4 (1.1)
	Missing	1 (0.3)
**Education**		
	No formal education	35 (9.6)
	Grade 7	13 (3.6)
	Grade 12	141 (38.7)
	Certificate	101 (27.8)
	Bachelor’s degree	59 (16.2)
	Higher degree	14 (3.9)
	Missing	1 (0.3)
**Employment status**		
	Unemployed	91 (25.0)
	Casually employed	69 (19.0)
	Self-employed	95 (26.1)
	Salaried employee	108 (29.7)
	Missing	1 (0.3)
**Economic status**		
	Poor	53 (14.6)
	Below average	70 (19.2)
	Average	190 (52.2)
	Above average	41 (11.3)
	Wealthy	10 (2.8)
**Residence**		
	Informal settlement	27 (7.4)
	House (owned)	54 (14.8)
	House (rented)	131 (36.0)
	Apartment (owned)	43 (11.8)
	Apartment (rented)	107 (29.4)
	Missing	2 (0.6)
**Household**		
	Living alone	35 (9.6)
	Living with 2 people	71 (19.5)
	Living with 3 people	93 (25.6)
	Living with 4 people	101 (27.8)
	Living with >5 people	62 (17.0)
	Missing	2 (0.6)
**Takes medication**		
	Yes	89 (24.5)
	No	274 (75.3)
	Missing	1 (0.3)
**Diabetic (told by doctor)**		
	Yes	45 (12.4)
	No	316 (86.8)
	Missing	3 (0.8)
**High blood sugar (told by doctor)**		
	Yes	46 (12.6)
	No	316 (86.8)
	Missing	2 (0.6)
**Risk of diabetes (told by doctor)**		
	Yes	51 (14.3)
	No	308 (84.6)
	Missing	4 (1.1)
**Weight**		
	Below average	42 (11.5)
	Average	283 (77.8)
	Above average	38 (10.4)
	Missing	1 (0.3)
**Body type**		
	Skinny	86 (23.6)
	Small	75 (20.6)
	Average	167 (45.9)
	Overweight	25 (6.9)
	Obese	11 (3.0)

^a^Percentages may not equal 100.0% due to rounding.

^b^“In South Africa, the term *Coloured* originated during the apartheid era to describe a distinct mixed ancestry community. The term is still used in South Africa as an official race group for census data and scientific research.” [28]

### Internal Reliability

The Cronbach alpha coefficients for each grouped variable are presented in Table 2. All grouped variables, except for medications, SMS original, and SMS were considered acceptable and reliable with a coefficient greater than the .7 limit.

For medication use, the mean interitem correlation of 0.307 was within the acceptable range of 0.3 to 0.5; however, the questions “I find it easy to manage my medications” and “I would like to be reminded when to take my medications” provided a very low value of 0.078. There was also a large inconsistency between the number of people who filled out the questions for this grouped variable (n=120) and the number of people who answered yes to the demographic question “Do you take any medications on a regular basis?” (n=89). Due to this discrepancy and the outlier from the interitem correlations, this grouped variable was not considered reliable and was removed from further analysis.

For SMS original, the mean interitem correlation of 0.262 was below the acceptable range. The question “I have access to a cell phone” was removed from the analysis, and the data were rerun to reveal a mean interitem correlation of 0.314, so a new variable titled SMS, containing the remaining 3 questions, was created and used for further analysis. Despite having a strong Cronbach alpha coefficient, it was determined that internet access did not appropriately address participants’ interest in receiving information, so it was removed from analysis as well.

### Mean Likert Scores

For diabetic risk factors, mean Likert scores closest to 5 represented the risk factors that participants were most interested learning about, whereas scores closest to 1 represented the risk factors that participants were least interested in. Diet and nutrition and physical activity had the highest mean Likert scores of 3.983 and 3.830, respectively, followed by alcohol use (3.314) and smoking (3.224). The lowest mean Likert score was 2.721 for hypertension. The mean Likert scores are presented with SDs in Table 3.

**Table 2:** Reliability coefficients of the grouped variables.

Outcome measure		Number of items	Test scale (alpha coefficient)	Mean interitem correlations (range)
**Diabetic risk factors**				
	Diet and nutrition	4	.75	0.429 (0.288-0.608)
	Physical activity	4	.756	0.437 (0.271-0.739)
	Smoking	4	.828	0.546 (0.435-0.662)
	Alcohol	4	.85	0.587 (0.440-0.798)
	Hypertension	4	.769	0.459 (0.327-0.747)
	Medication use	4	.636	0.307 (0.078-0.581)
**Information delivery**				
	Television	3	.825	0.609 (0.538-0.725)
	Radio	3	.867	0.686 (0.645-0.761)
	Newspaper	3	.906	0.763 (0.700-0.820)
	SMS^a^ original	4	.582	0.262 (0.005-0.405)
	SMS	3	.577	0.314 (0.247-0.405)
	WhatsApp	4	.747	0.444 (0.200-0.829)
	Internet access	3	.795	0.561 (0.444-0.791)
	Email	4	.768	0.453 (0.320-0.652)
	Mobile apps	4	.81	0.516 (0.347-0.720)
	Social media	4	.793	0.486 (0.296-0.736)
	Face to face	4	.78	0.476 (0.183-0.629)

^a^SMS: short message service.

**Table 3:** Mean Likert scores for diabetic risk factors and information delivery methods.

Outcome variables		n	Mean Likert score (SD)
**Diabetes risk factors**			
	Diet and nutrition	361	3.983 (0.721)
	Physical activity	362	3.830 (0.734)
	Smoking	361	3.224 (1.116)
	Alcohol	360	3.314 (1.159)
	Hypertension	360	2.721 (1.028)
**Information delivery methods**			
	Television	360	3.995 (0.933)
	Radio	363	3.318 (1.060)
	Newspaper	359	3.320 (1.172)
	SMS^a^	359	3.356 (0.927)
	WhatsApp	359	3.844 (0.917)
	Email	361	2.757 (1.076)
	Mobile apps	361	2.747 (1.044)
	Social media	363	3.009 (1.070)
	Face to face	364	2.732 (1.086)

^a^SMS: short message service.

For information delivery methods, mean Likert scores closest to 5 represented the interventions that participants were most receptive toward using, whereas scores closest to 1 represented the risk factors that participants were least receptive toward. Television and WhatsApp had the highest mean Likert scores of 3.995 and 3.844, respectively, followed by SMS (3.356), newspaper (3.320), radio (3.318), and social media (3.009). The lowest mean Likert scores were face-to-face interactions (2.732), mobile apps (2.747), and email (2.757). The mean Likert scores are presented with SDs in Table 3.

### Correlations

To detect relationships between diabetic risk factors and information delivery methods, nonparametric correlations were explored with Spearman Rho correlation coefficients. The strongest correlations were between the risk factor diet and nutrition and the information delivery methods WhatsApp and television, with moderate correlation coefficients of 0.338 (*P*<.001) and 0.312 (*P*<.001), respectively. Physical activity also had weaker correlations with the same information delivery methods; WhatsApp had a correlation coefficient 0.243 (*P*<.001) and television had a correlation coefficient of 0.294 (*P*<.001).

Diet and nutrition also had weak correlation coefficients with email (0.145; *P*=.006) and face-to-face interactions (−0.258; *P*<.001), whereas physical activity also had a weak correlation with email (0.157; *P*=.003). Smoking also had weak correlations with television (0.152; *P*=.004), radio (0.190; *P*<.001), newspaper (0.210; *P*<.001), mobile apps (0.160; *P*=.002), and social media (0.116; *P*=.03). Alcohol only had 1 weak correlation with newspaper (0.174; *P*=.001), whereas hypertension had 2 weak correlations with newspaper (0.161; *P*=.002) and mobile apps (0.107; *P*=.04). A complete list of all correlation coefficients is presented in Table 4.

**Table 4:** Spearman Rho correlation coefficients for risk factors and delivery methods.

Information delivery methods	Diabetic risk factors														
	Diet and nutrition			Physical activity			Smoking			Alcohol			Hypertension		
	ρ	*P* value^a^	N	ρ	*P* value	N	ρ	*P* value	N	ρ	*P* value	N	ρ	*P* value	N
Television	*0.312* ^b^	<.001	361	*0.294*	<.001	362	*0.152*	.004	361	0.091	.08	360	0.045	.39	360
Radio	0.050	.35	361	0.060	.25	362	*0.190*	<.001	361	0.049	.35	360	0.072	.17	360
Newspaper	0.009	.86	361	0.025	.64	362	*0.210*	<.001	361	*0.174*	.001	360	*0.161*	.002	360
SMS^c^	0.006	.91	361	0.048	.36	362	0.103	.05	361	0.058	.27	360	0.027	.61	360
WhatsApp	*0.338*	<.001	361	*0.243*	<.001	362	-0.081	.12	361	0.019	.71	360	-0.048	.36	360
Email	*0.145*	.006	361	*0.157*	.003	362	0.065	.22	361	0.057	.28	360	0.070	.19	360
Mobile apps	−0.050	.35	361	0.053	.31	362	*0.160*	.002	361	−0.020	.71	360	*0.107*	.04	360
Social media	−0.042	.42	361	−0.015	.78	362	*0.116*	.03	361	0.005	.92	360	0.002	.99	360
Face to face	*−0.258*	<.001	361	−0.079	.13	362	0.069	.19	361	−0.098	.06	360	0.029	.58	360

^a^Two-tailed significance.

^b^Satistically significant correlations are presented in *italics*.

^c^SMS: short message service.

## Discussion

### Principal Findings

This research described the studied commuter population from the City of Johannesburg and their perceptions toward diabetic risk factors and information delivery methods. Although the national prevalence of diabetes in South Africa is 5.5%, the prevalence in this studied commuter population was greater than twice as high, at 12.4% [1], which reinforces the need for risk factor education, especially in the studied commuter population.

This study also provided the commuter population in Johannesburg with an opportunity for input, to ensure that new diabetic mHealth interventions have the greatest potential for acceptability and usability with the targeted end users. When the mHealth interventions were investigated for relationships with diabetes risk factors, WhatsApp showed the strongest correlations. WhatsApp had a moderate correlation with diet and nutrition as well as a weaker correlation with physical activity. This suggests that the most feasible mHealth intervention for diabetic risk factor education should feature WhatsApp to provide content focusing on diet and nutrition as well as physical activity.

Many of the other mHealth interventions had correlations with risk factors as well, and although these relationships were not as robust as the WhatsApp correlations, they were still statistically significant. This introduces the prospect of a multifaceted mHealth approach that does not solely rely on 1 information delivery method, and this combined approach could allow for a tailored experience, where end users may interact with different platforms to obtain information. This varied approach has already been implemented by other successful mHealth platforms in South Africa. Some interventions use messaging services as the backbone, but by offering information across different platforms, they removed barriers to access while also providing users with the possibility for enhanced interactions [12].

For traditional information delivery methods, television had a moderate correlation with diet and nutrition as well as weaker correlations with physical activity, whereas all of the others except for face-to-face interactions also had weak but statistically significant correlations. These findings suggest that the combined approach can also be extended to traditional media as participants were receptive toward receiving information on these platforms. Traditional media, however, is often more expensive than mHealth interventions and only offers 1-way communications; so, they should be used as a way to create awareness and push users toward the interactive mHealth platforms [29].

The digital landscape is very dynamic and always changing, so certain platforms will come and go based on network capabilities and consumer demand. The combined mHealth approach also ensures that end users are engaged on platforms they are comfortable with now, while also introducing new platforms to ensure that the interventions stay current and in line with digital trends [30]. Another advantage of mHealth interventions is the possibility of tailoring messages to specific subdemographics of interest [29]. Although the exploration of subdemographics is beyond the scope of this study, future research should focus on defining higher risk subdemographics within the studied commuter population and creation of specific messages catered to their specific circumstances.

### Limitations

Our study presented some limitations. This was a new survey that had not been validated, and convenience sampling of the targeted commuter population may have introduced a selection bias. The survey was only offered in written English, which may have excluded some of the population. The background statistics about socioeconomic status; being told by a doctor they were diabetic, had high blood sugar, or were at risk of diabetes; as well as the questions about body weight and type were all self-reported by the participants, and no measurements were taken to validate these statements. This use of nonstandardized questions may affect the external validity of the survey.

Of the 400 surveys that were collected, 36 surveys had at least five (6.7%) questions left blank and were considered spoiled. These spoiled surveys were not used in the data analysis, and no further tests were done on them. There were also discrepancies between the number of people who answered yes to the question about regular medication use in the demographic section and the diabetic risk factor section (120 vs 89), which may have been caused by inadequately defining the words *regular* and *medication* in the survey. This, combined by the lack of internal reliability of this variable, prevented further analysis of medication use. It was also discovered that the section pertaining to internet access in the information delivery systems did not appropriately address participants’ interest in receiving information, so it was removed from analysis as well.

### Conclusions

The prevalence of diabetes was twice as high in the studied commuter population than the national average, and this reinforces the need for innovative interventions that focus on prevention and management of diabetes. The South African mHealth Strategy 2015-2019 provides a backbone for creating mHealth interventions to address the diabetes epidemic; however, the body of evidence is not great enough to provide a tested blueprint for these interventions. The aim of this study was to investigate the feasibility and acceptability of mHealth interventions that increase awareness of diabetic risk factors among the studied commuter population in the City of Johannesburg to provide a starting point for future mHealth interventions.

Building from the combined-intervention approach found in the other successful mHealth programs and the statistically significant results in this study, the most practical mHealth intervention disseminating diabetes risk factor information to the studied commuters in the City of Johannesburg has been identified. This intervention should focus on WhatsApp messaging but offer content across other mHealth and traditional platforms to remove barriers to access and enhance the user experience. The content should emphasize the primary risk factors such as diet and nutrition as well as physical activity while also incorporating information on secondary risk factors such as smoking, alcohol use, and hypertension.

## Appendix

Multimedia Appendix 1 Data collection survey.

## Abbreviations

mHealth: mobile health
NCD: noncommunicable disease
SMS: short message service
