# Supplementary material for: Acceptability of Mobile Health Interventions to Increase Diabetic Risk Factor Awareness Among the Commuter Population in Johannesburg: Descriptive Cross-Sectional Study
Source: JMIR Diabetes. 2019 Sep 20;4(3):e12600. doi: 10.2196/12600 (PMC6779026; doi:10.2196/12600)
Supplement: Multimedia Appendix 1 [file diabetes_v4i3e12600_app1.pdf]

Acceptability of mhealth Interventions to Increase Diabetic Risk Factor

Awareness among the Commuter Population in Johannesburg

INSTRUCTIONS:

Please answer all questions.
Please indicate your answer with an 'X'.
Please indicate your consent with the survey below:

EXAMPLE of how to complete this questionnaire:

Your gender?
If you are female:

Male	1
Female	~~2~~ X

Section A – Demographics

This section of the questionnaire refers to background or biographical information. Although we are aware of the sensitivity of the questions in this section, the information will allow us to compare groups of respondents. Once again, we assure you that your response will remain anonymous. Your co-operation is appreciated.

1. Gender

Male	1
Female	2

2. Age (in complete years)

3. Ethnicity

Black	1
White	2
Coloured	3
Indian or Asian	4
Other	5

4. Marital status

Single	1
Living with partner	2
Married	3
Divorced/separated	4
Widowed	5

5. Highest level of education completed?

No formal education	1
Grade 7	2
Grade 12	3
Certificate	4
Bachelor's degree	5
Higher degree	6

6. What is your employment status?

Unemployed	1
Casually employed	2
Self-employed	3
Salaried employee	4

7. How would you describe your economic status?

Poor	1
Below average	2
Average	3
Above average	4
Wealthy	5

8. Please describe the place you reside

Informal settlement	1
House (owned)	2
House (rented)	3
Apartment (owned)	4
Apartment (rented)	5

9. How many people live in your household (including yourself)?

Live alone	1
2	2
3	3
4	4
More than 5	5

10. Do you take any medications on a regular basis?

Yes	1
No	2

11. Have you ever been told by a doctor that you have diabetes?

Yes	1
No	2

12. Have you ever been told by a doctor that you have high blood sugar?

Yes	1
No	2

13. Have you ever been told by a doctor that you are at risk of diabetes?

Yes	1
No	2

14. Please describe your weight:

Below average	1
Average	2
Above average	3

15. Please describe your body type:

Skinny	1
Small	2
Average	3
Overweight	4
Obese	5

Section B- Diabetic Risk Factors

This section explores your attitude and perceptions toward different diabetic risk factors.

To what extent do you agree with each of the following statements. Please indicate your answer using the following 5-point scale where: 1= Never
2= Rarely
3= Sometimes
4= Often
5= Always

	Strongly disagree	Disagree	Neutral	Agree	Strongly agree
**Diet and Nutrition**					
1. Overall I feel that I eat a healthy diet.	1	2	3	4	5
2. I know which foods are healthy and which are unhealthy.	1	2	3	4	5
3. I would like more information on how to eat a healthier, balanced diet.	1	2	3	4	5
4. I would like to receive healthy recipes that are easy to make.	1	2	3	4	5
**Physical Activity**					
1. I am physically active for at least one hour, three times a week.	1	2	3	4	5
2. Physical activity is important for healthy aging.	1	2	3	4	5
3. I would like more information about the benefits of physical activity.	1	2	3	4	5
4. I would like more information about how to become more physically active.	1	2	3	4	5
**Secondary Risk Factors – Smoking, Alcohol and Hypertension**					
1. I smoke cigarettes on a daily basis.	1	2	3	4	5
2. People around me smoke cigarettes on a regular basis?	1	2	3	4	5
3. I know the health risks associated with cigarette smoke.	1	2	3	4	5
4. I would like more information on the risks of smoking.	1	2	3	4	5
5. I drink alcohol on a regular basis.	1	2	3	4	5
6. When I drink alcohol, it is to get drunk.	1	2	3	4	5
7. I know the risks of long term alcohol use.	1	2	3	4	5
8. I would like more information on the risks of alcohol.	1	2	3	4	5
9. I have been told by a doctor that I have high blood pressure.	1	2	3	4	5
10. I have been told by a doctor that I am at risk of getting high blood pressure (hypertension).	1	2	3	4	5
11. I understand the risks associated with high blood pressure.	1	2	3	4	5
12. I would like more information about the risks of high blood pressure.	1	2	3	4	5
**Medications**<br>**Please only answer this section if you take medications regularly**					
1. I find it easy to manage my medications.	1	2	3	4	5
2. Sometimes I forget to take my medications.	1	2	3	4	5
3. I would like to be reminded when to take my medications.	1	2	3	4	5
4. I would like more information on the medications that I take.	1	2	3	4	5

Section C- Information Delivery Methods

This section explores your attitude and perceptions toward different information delivery methods.

How often do you do you find yourself in each of the following situations? Please indicate your answer using the following 5-point scale where: 1= Never
2= Rarely
3= Sometimes
4= Often
5= Always

	Never	Rarely	Sometimes	Often	Always
**Traditional Media**					
1. I have access to a television.	1	2	3	4	5
2. I watch television as a source of news and information.	1	2	3	4	5
3. I would like more information that could improve my health from the television.	1	2	3	4	5
4. I have access to a radio.	1	2	3	4	5
5. I listen to the radio as a source of news and information.	1	2	3	4	5
6. I would like more information that could improve my health from the radio	1	2	3	4	5
7. I have access to a daily newspaper.	1	2	3	4	5
8. I read the paper as a source of news and information.	1	2	3	4	5
9. I would like more information that could improve my health from the newspaper.	1	2	3	4	5
**Cell Phones- SMS Texts**					
1. I have access to a cell phone.	1	2	3	4	5
2. I use SMS text to communicate with people.	1	2	3	4	5
3. I have used SMS text messages to talk to people about my health.	1	2	3	4	5
4. I would like to get SMS text messages that could improve my health.	1	2	3	4	5
**Cell Phones- WhatsApp**					
1. I have access to a WhatsApp on a cell phone.	1	2	3	4	5
2. I use WhatsApp to communicate with people.	1	2	3	4	5
3. I have used WhatsApp messages to talk to people about my health.	1	2	3	4	5
4. I would like to get WhatsApp messages that could improve my health.	1	2	3	4	5
**Internet Access**					
1. I have access to the internet on a phone.	1	2	3	4	5
2. I have access to the internet on a tablet.	1	2	3	4	5
3. I have access to the internet on a computer.	1	2	3	4	5
**Email**					
1. I have an email address.	1	2	3	4	5
2. I use email as a source of news and information.	1	2	3	4	5
3. I have used email messages to talk to people about my health.	1	2	3	4	5
4. I would like to get email information that could improve my health.	1	2	3	4	5
**Mobile Apps**					
1. I have access to mobile apps on a phone or tablet	1	2	3	4	5
2. I use mobile apps as a source of news and information.	1	2	3	4	5
3. I have used mobile apps to talk to people about my health.	1	2	3	4	5
4. I would like to get information from mobile apps that could improve my health.	1	2	3	4	5
**Social Media**					
1. I use social media sites like Facebook and Twitter.	1	2	3	4	5
2. I use social media sites as a source of news and information.	1	2	3	4	5
3. I have used social media to talk to people about my health.	1	2	3	4	5
4. I would like to use social media interactions that could improve my health.	1	2	3	4	5
**Face-to-Face Interactions**					
1. I interact regularly with people in the community.	1	2	3	4	5
2. I get my news and information from talking directly to people in the community.	1	2	3	4	5
3. I have talked to people in the community about my health.	1	2	3	4	5
4. I would like more information from people in the community that could improve my health.	1	2	3	4	5
